# The Pro-Angiogenic Potential of Periodontal Ligament Stem Cells and Dental Pulp Stem Cells: A Comparative Analysis

**DOI:** 10.3390/cells14120864

**Published:** 2025-06-08

**Authors:** Ilaria Roato, Clarissa Orrico, Sara Meinardi, Riccardo Pedraza, Alessandro Mosca Balma, Giacomo Baima, Tullio Genova, Mario Aimetti, Federico Mussano

**Affiliations:** 1 Bone and Dental Bioengineering Laboratory, CIR Dental School, Department of Surgical Sciences, University of Turin, 10126 Turin, Italy; ilaria.roato@unito.it (I.R.); clarissa.orrico@unito.it (C.O.); riccardo.pedraza@unito.it (R.P.); alessandro.moscabalma@unito.it (A.M.B.); 2 Department of Mechanical and Aerospace Engineering, Politecnico di Torino, Corso Duca degli Abruzzi 24, 10129 Turin, Italy; 3 Department of Life Sciences and Systems Biology, University of Turin, Via Accademia Albertina 13, 10123 Turin, Italy; sara.meinardi@edu.unito.it (S.M.); tullio.genova@unito.it (T.G.); 4 Institute of Sciences and Technologies for Sustainable Energy and Mobility, National Council of Research, Strada delle Cacce 73, 10135 Turin, Italy; 5 Periodontology Unit, CIR Dental School, Department of Surgical Sciences, University of Turin, 10126 Turin, Italy; giacomo.baima@unito.it (G.B.); mario.aimetti@unito.it (M.A.)

**Keywords:** periodontal ligament stem cell, dental pulp stem cell, angiogenesis

## Abstract

The role of periodontal ligament stem cells (PDLSCs) and dental pulp stem cells (DPSCs) in stimulating angiogenesis has been reported, but their angiogenetic potential has not been directly compared. In this work, paired PDLSCs and DPSCs, i.e., derived from the same donor, were tested for their immunophenotype and multi-differentiation capabilities, with particular emphasis on their pro-angiogenic activity. Flow cytometry was utilized to study the expression of mesenchymal stem cell, pericyte, and endothelial markers, while gene expression was evaluated through real-time PCR. The angiogenic potential was assessed recurring to tubulogenesis assay, co-cultures with Human Microvascular Endothelial Cell (HMEC-1), and VEGF-A quantification. The immunophenotype of DPSCs and PDLSCs was different in CD146+ and CD31+ cell subsets, but both cell types promoted HMEC-1 tubulogenesis in vitro. Consistently, VEGF-A gene expression level and its quantification in cell-conditioned media of PDLSCs and DPSCs was comparable between them, and both promoted the formation of vessel-like structures, when co-cultured with HMEC-1 cells. All together, these results showed the heterogeneity of PDLSCs and DPSCs, which are constituted of different cellular subsets, likely modulated by the microenvironmental cues. PDLSCs and DPSCs showed comparable pro-angiogenic activity, enhanced by the contemporary expression of angiogenic and chemotactic factors.

## 1. Introduction

Ubiquitous in the human body, mesenchymal stem cells, also known as mesenchymal stromal cells, (MSCs) are multipotent, non-hematopoietic stem cells, indispensable for the homeostatic maintenance and regeneration of a large variety of tissues, owing, by definition, to their capacity of differentiating into multiple histotypes without losing their self-renewal properties [1]. MSCs have been further defined through the compresence of three capabilities: (a) adhesion to tissue culture plastic; (b) adipo-, chondro-, and osteo-differentiation potential; and (c) the positivity (≥95%) for the surface antigens CD105, CD90, and CD73 along with the negativity (≤2%) for CD45, CD34, CD14 or CD11b, CD79α, or CD19 and HLA-DR [2]. The oral cavity is known to harbor a variety of MSCs [3], which exhibit a unique feature, i.e., the neuro-ectomesenchymal origin. During early embryogenesis, indeed, neural crest cells (NCCs), upon migration to develop branchial arches [4], give rise to the precursors of cranial cartilages and bones, generating most of craniofacial tissues, including teeth [5,6,7,8]. Hence, MSCs harvested from teeth derive from NCCs, of which they retain some peculiar aspects [9,10].

Among dental MSCs, periodontal ligament (PDL)-derived stem cells (PDLSCs) and dental pulp stem cells (DPSCs) have been the focus of extensive research, aiming mostly at tissue regeneration. PDLSCs were shown to generate periodontal attachment-like structures when transplanted into immune-compromised mice [11] and in rat model of periodontitis [12], proving useful for periodontal regeneration in animal models [13,14,15,16] and human clinical trials [17]. Likewise, DPSCs were used for ectopic regeneration of pulp-dentinal complex in SCID mice [18] and in humans for periodontal bone regeneration [19]. While, as Huang et al. [20] stated five years ago, with unaltered validity, “we are still far short of reaching the standard of complete vascularized and innervated pulp regeneration with newly formed tubular dentin in all types of teeth”, recent encouraging reports hold promise for future procedures based on smart scaffold systems supporting both cell survival and commitment in vivo [21].

PDLSCs and DPSCs represent distinct cellular populations of the same tooth, but in distinct compartments [22]. They can differentiate in different tissues, due to the stemness characteristics of the cells. In particular, some authors have reported that PDLSCs proliferate faster than DPSCs, but the latter seem to have a better osteogenic potential [23]; indeed, in another work, DPSCs are suggested for the regeneration of periodontal bone tissue [24]. The pro-angiogenic activity of PDLSCs and DPSCs has been investigated by comparing them to other oral cavity MSCs [25,26] or MSCs deriving from other tissues, such as bone marrow or adipose tissue [27,28]. None of the studies revealed the best candidate, nor compared PDLSCs and DPSCs in terms of angiogenic potential. Angiogenesis is fundamental for the success of bone regeneration [29]. DPSCs and PDLSCs can exert their angiogenic potential indirectly, through the release of a wide variety of pro-angiogenic factors [30], or directly, by differentiating into endothelial cells (ECs) [31]. Alternatively, DPSCs have the capacity to work as pericytes supporting the vascular structures and inducing a higher vessels’ maturation when co-cultured with endothelial cells both in vitro and in vivo [27]. PDLSCs also possess a pericyte-like supporting function which can significantly enhance the angiogenic process in vivo [32]. In the present study, the authors analyzed paired, i.e., deriving from the same donor, PDLSCs and DPSCs (healthy teeth) to investigate their immunophenotype and pro-angiogenic potential.

## 2. Materials and Methods

### 2.1. Isolation and Expansion of PDLSCs and DPSCs

The PDLSCs and DPSCs were isolated from the mandibular wisdom teeth of 5 healthy donors, in accordance with ethical principles and the Helsinki declaration. The study was approved by the local Ethics Committee (n° 0107683) and subjects signed an informed consent form. Briefly, for harvesting PDLSCs, the dental root was scratched, and the fragments of tissue were digested for 10 min with a solution of 1 mg/mL dispase II (PluriSTEM™, Merk, Rahway, NJ, USA) and 3 mg/mL collagenase I (Sigma Aldrich, Burlington, MA, USA). For DPSCs, the tooth crown was cut, and the dental pulp was removed gently and digested as above. The tissues thus treated were washed and seeded in α-MEM with 15% fetal bovine serum (FBS), 5% penicillin/streptomycin, and 5 ug/mL gentamicin (Life Technologies, Carlsbad, CA, USA). Every 5 days, the medium was refreshed, monitoring the cellular growth until they reached the confluence. Cells were then detached, counted, and expanded in a medium containing α-MEM + Glutamax, with 5% of human platelet lysate (hpl) (IsoCell Growth, Euroclone, Pero, Italy) and 1% penicillin/streptomycin.

Conditioned medium (CM) of both PDLSCs and DPSCs, at passage 3, was collected after 72 h of incubation; the culture supernatant was centrifuged at 1500 rpm for 5 min, then collected, filtered, and stored at −80°C until use.

### 2.2. Immunophenotype Analysis

The immunophenotypes of DPSCs and PDLSCs were studied for some in vitro cell culture passages, through flow cytometry by labeling with monoclonal fluorochrome-conjugated antibodies against the following surface antigens: CD105, CD73, CD90, CD44, CD146, CD31, CD45, and EpCam1. CD105 PE (Invitrogen, Camarillo, CA, USA), CD73 FITC, CD44 FITC, CD45 PerCP, CD31PE, CD146APC (Miltenyi Biotech, Bergisch Gladbach, Germany), and CD90 PerCP (Biolegend, San Diego, CA, USA). As a control, unstained cells were examined. Data were obtained on a MACsQuant 10 and computed with MACsQuantify software (Miltenyi Biotech, Bergisch Gladbach, Germany). The data are presented as percentages of cells expressing specific markers (mean ± SD).

### 2.3. Assays of Multi-Differentiation Capability

To study DPSCs and PDLSC multi-differentiating capabilities, the cells were cultured in the specific media. DPSCs and PDLSCs were cultured in osteogenic medium (OM), constituted by α-MEM, with 10% FBS, 50 μg/mL ascorbic acid, 10^−8^ M dexamethasone, and 10 mM beta-glycerophosphate (Sigma Aldrich, Burlington, MA, USA) for 30 days. Then, Von Kossa staining was performed to evaluate the mineralized matrix. Adipogenic and chondrogenic media were purchased from Miltenyi (Miltenyi Biotech, Bergisch Gladbach, Germany) and cells were cultured for 21 days. Oil red O staining allowed the lipid droplets in adipocytes to be visualized, and Aggrecan (ACAN) immunofluorescence staining enabled the detection of chondrocyte micro-masses.

### 2.4. RNA Isolation and Real-Time

RNA from both DPSCs and PDLSCs was extracted at p1 through the RNeasy Kit according to manufacturer’s instructions (Qiagen, Venlo, The Netherlands); a measure of 1 µg of RNA was retrotranscribed to single-stranded cDNA by the High-Capacity cDNA Reverse Transcription Kit from Applied Biosystems (Thermo Fisher Scientific, Carlsbad, CA, USA). The mRNA expression of the following genes was assessed: SOX2, OCT3-4, NANOG, RUNX2, SOX9, PPARγ, and β-actin, utilizing primers previously published [33]. Primer sequences for CXCR4 were FW 3′-CTCGCCTTCATCAGTCTGGA-5′ and REV 3′-TCATCTGCCTCACTGACGTT-5′; for COLL-2A1 FW 3′-TCTACCCCAATCCAGCAAAC-5′ and REV 3′-GTTGGGAGCCAGATTGTCAT-5′, real-time PCR was performed with Luna^®^ Universal qPCR Master Mix (New England BioLabs, Ipswich, MA, USA) in the CFX96 system (Bio-Rad, Hercules, CA, USA). For VEGF and CXCL12/SDF-1 (stromal-derived factor 1), we utilized primers designed by Biorad (PrimePCR^TM^ SYBR^®^ Green Assay) and iTaq Universal SYBR Green (Biorad, Hercules, CA, USA). The amplification protocol foresees 40 cycles with an annealing temperature of 60 °C. The expression of β-actin was chosen to normalize gene expression data and the 2−ΔΔCt method was used for the quantitative analysis using CFX Manager software (Bio-Rad, Hercules, CA, USA). Gene expression data are presented as mean ± SEM.

### 2.5. In Vitro Tube Formation Assay

The tubulogenic capability of HMEC-1 (1.0 × 10^4^ cells/well) (CLS Cell Lines Service GmbH, Eppelheim, Germany) was studied according to a previously published protocol [34]. Briefly, HMEC-1 were plated on growth factor-reduced Matrigel (Corning, NY, USA) in μ-plate 96-well 3D (IBIDI GmBh, Gräfelfing, Germany), utilizing conditioned medium of DPSCs and PDLSCs at 100% and 50% with α-MEM. Cell organization in Matrigel was acquired after 13 h using a Nikon Eclipse Ti E microscope using a Nikon Plan 10×/0.10 objective (Nikon Instruments, Amsterdam, The Netherlands). Three independent experiments were performed for each experimental condition. The analysis of tubulogenesis was performed using the angiogenesis analyzer tool of ImageJ 2.9 developed by Gilles Carpentier [35], considering several parameters: (a) nodes are pixels with 3 neighbors represented as a circular dot; (b) junctions correspond to nodes or groups of fusing nodes; (c) segments are elements delimited by 2 junctions; (d) isolated elements are binary lines that are not branched; (e) master segments consist of pieces of tree (complex structure made of nodes and segments) delimited by 2 junctions, none exclusively implicated with one branch, called master junctions; and (f) master junctions are junctions linking at least three master segments.

### 2.6. VEGF Dosage in PDLSC and DPSC Supernatants

In supernatants of PDLSC and DPSCs utilized for CM, the VEGF secretion was assessed by enzyme-linked immunosorbent assay (ELISA) with a human VEGFa ELISA kit, according to manufacturer’s instructions (Sigma-Aldrich, Burlington, MA, USA).

### 2.7. Immunofluorescence Staining of HMEC Co-Cultured with PDLSCs and DPSCs

Co-cultures of HMEC-1 (seeded 1.25 × 10^4^/well) with PDLSCs and DPSCs (seeded 3.75 × 10^4^/well) were performed on 8-well Chamber slides (Life Technologies, Carlsbad, CA, USA) in endogrow, supplemented with 5% FBS, 5 ng/mL rhVEGF, 5 ng/mL rhEGF, 5 ng/mL rhFGF, 15 ng/mL rhIGF-1, 1 µg/mL Hydrocortisone hemi-succinate, 0.75 U/mL heparin sulfate, 10 mM L-glutamine, and 50 µg/mL ascorbic acid, according to manufacturer’s instructions (Merck KGaA, Darmstadt, Germany). After 21 days, cells were fixed with 4% of paraformaldehyde solution, then washed with phosphate saline buffer (Euroclone S.p.A, Pero, Italy). After permeabilization with TBS 1X (Life Technologies, Carlsbad, CA, USA) containing 0.5% Triton X-100 (Bio-Rad Laboratories, Segrate, Italy) for 1 min, IMAGE-IT FX (Life Technologies, Carlsbad, CA, USA) signal enhancer was applied for 45 min to saturate and reduce non-specific signals. CD31PE (Miltenyi Biotech, Bergisch Gladbach, Germany) incubation was performed for 1 h, and the cells were then washed. Alexa Fluor 488 Phalloidin (Cell Signaling Technology, Danvers, MA, USA) incubation was performed for 45 min, then the cells were washed. Nuclei were marked with DAPI (Merck KGaA, Darmstadt, Germany). Images were acquired using a Nikon Eclipse Ti-E microscope with Nikon Plan 10×/0.10 objective.

### 2.8. Statistical Analysis

A descriptive analysis was performed with the data presented via mean ± standard error of mean (±SEM). The Shapiro–Wilk test was used to test for normality, then a one-way ANOVA with a multiple comparison was performed. In case that the data collected were nonparametric, differences among groups were analyzed with a Kruskal–Wallis test. All data were analyzed in Graphpad Prism 7 software (GraphPad Software, Inc., La Jolla, CA, USA). A 0.05 level was set as significance. 

## 3. Results

### 3.1. The Immunophenotype of DPSCs and PDLSCs Differs in the CD146+ Cell Subset

**Figure 1:**
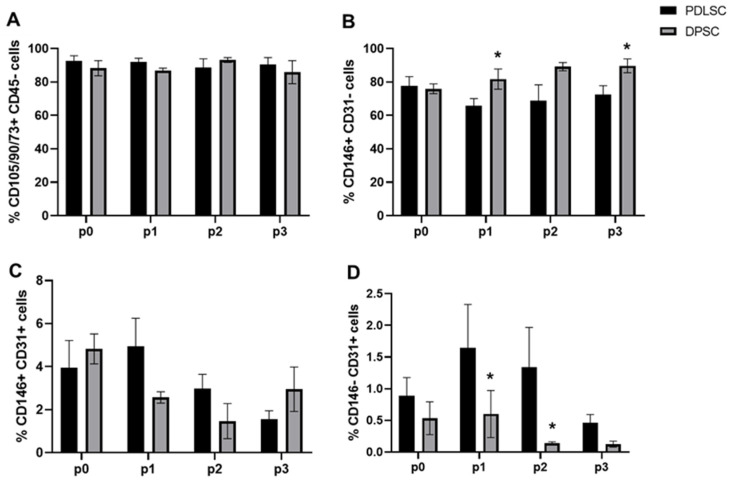
Immunophenotype analysis. The percentage of mesenchymal stem cells expressing the canonical markers CD73, CD90, and CD105 was high and comparable during the culture passages for both PDLSCs and DPSCs (**A**). The CD146 + CD31- cell subset increased in DPSCs at p1 and p3: * *p* < 0.05 (**B**). The double positive CD146/CD31 cell subset did not show significant variation (**C**), and the CD31 + CD146- cells were higher in PDLSCs than in DPSCs during the passages: * *p* < 0.05 (**D**).

We investigated whether the immunophenotype of DPSCs and PDLSCs was different, and whether it could be modified during the culture passages. Mesenchymal stem cells, expressing the canonical markers CD73/CD90/CD105 + CD45-, were highly expressed and were maintained during the culture time (Figure 1A). We analyzed more in detail the subset of cells expressing CD146 (pericyte marker) and CD31 (endothelial marker), showing that the subset of CD146 + CD31- cells was significantly increased in DPSCs compared to PDLSCs at p1 and p3 (* *p* < 0.05, Figure 1B). The CD146+ CD31+ cellular subset was higher soon after isolation (p0) in both DPSCs and PDLSCs, but it decreased during the passages (Figure 1C). The CD31+ CD146- cells were higher in PDLSCs compared to DPSCs (Figure 1D).

### 3.2. The Multi-Differentiation Capability of PDLSCs and DPSCs

**Figure 2:**
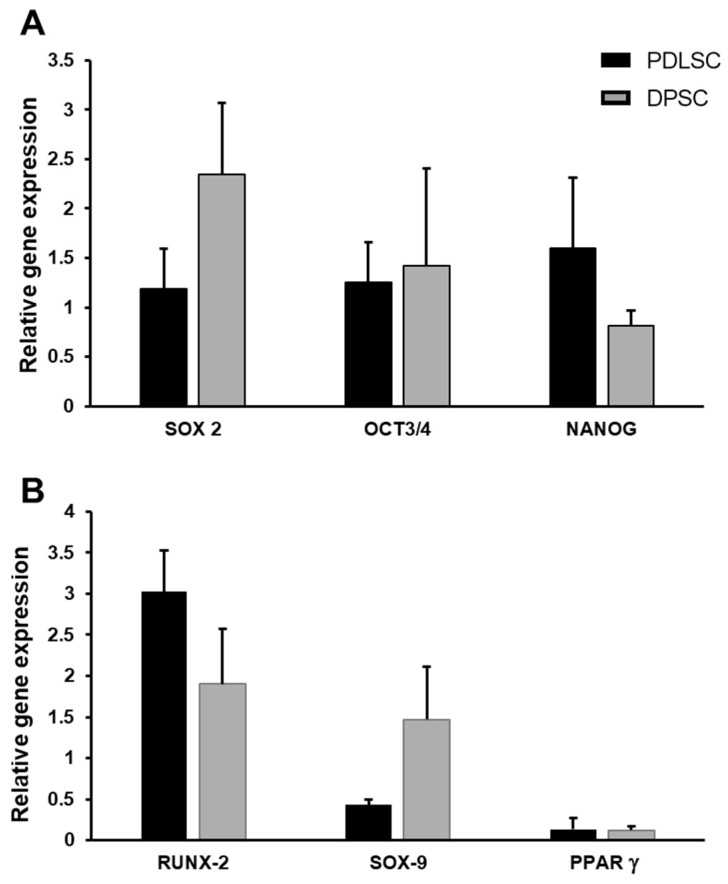
Gene expression analysis of PDLSCs and DPSCs. At basal condition, PDLSCs and DPSCs expressed comparable levels of stemness genes (**A**). The expression of RUNX-2, SOX-9 and PPARγ did not show significant differences among PDLSC and DPSC (**B**).

The expression levels of the stemness genes SOX2, OCT3/4, and NANOG were evaluated, showing no significant difference between PDLSCs and DPSCs at p1 (Figure 2A). Since the multi-differentiation capability is one of the main characteristics of all MSCs, we also evaluated the expression of the master genes of osteogenesis (RUNX-2), chondrogenesis (SOX-9), and adipogenesis (PPARγ) in PDLSCs and DPSCs, cultured in the basal medium, to exclude the presence of specific commitment in our cells. In general, a high expression of RUNX-2 was found in both PDLSCs and DPSCs, while PDLSCs seemed to be less chondrogenic than DPSCs, and both showed low expression levels of PPARγ (Figure 2B).

**Figure 3:**
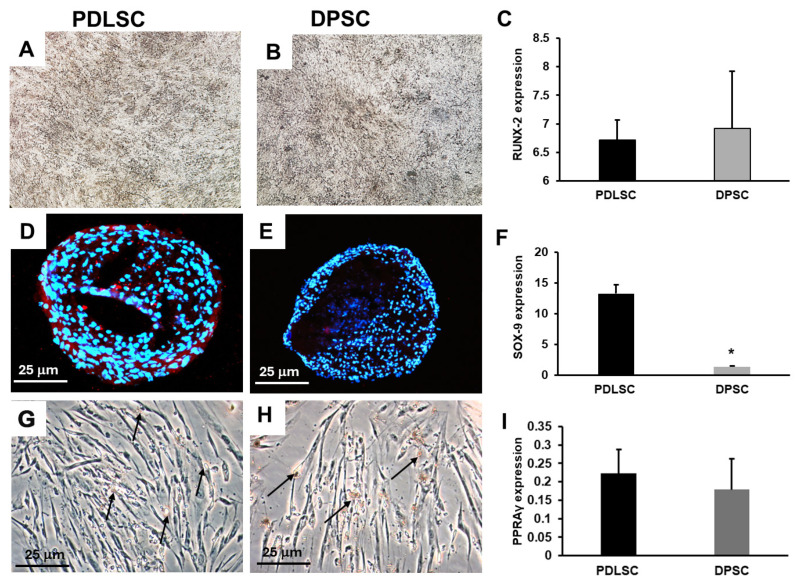
Multi-differentiating capability of PDLSCs and DPSCs. Osteogenic medium induced osteoblast differentiation with the release of mineralized matrix, visualized as dark granules (**A**,**B**), and expression of RUNX-2 (**C**). The chondro-differentiation medium induced chondrocyte masses, positive for aggrecan (red) in PDLSCs and less in DPSCs (**D**,**E**), which also expressed less SOX-9 than PDLSCs: * *p*< 0.05 (**F**). Adipo-differentiation medium induced the formation of few Oil red O adipocytes in both PDLSCs and DPSCs (**G**,**H**) and they expressed comparable and low levels of PPARγ (**I**).

Then, we tested the ability of PDLSCs and DPSCs to differentiate into osteoblasts, chondrocytes, and adipocytes when maintained in the specific differentiation cell culture media. PDLSCs and DPSCs showed a comparable ability to release matrix, as shown by Von Kossa staining (Figure 3A,B), and expressed comparable levels of RUNX-2 (Figure 3C). The ability to form aggrecan+ chondro-masses was lower in DPSCs than in PDLSCs (Figure 3D,E) and SOX9 was significantly less expressed in DPSCs than in PDLSCs, *p* < 0.05 (Figure 3F), as well as COLL-2A1 (Figure S1). The formation of adipocytes containing oil droplets was low (Figure 3G,H) as well as the level of PPARγ expression for both PDLSCs and DPSCs (Figure 3I).

### 3.3. PDLSCs and DPSCs Show Comparable Angiogenic Potential

**Figure 4:**
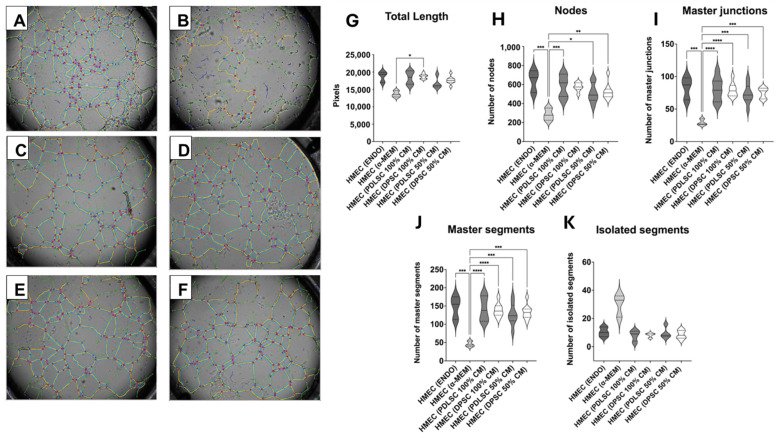
Tubulogenesis assay. Vessel-like structures are visible in HMEC-1 in endogrow medium (**A**), in α -MEM medium (**B**), in PDLSC 100% CM (**C**), in DPSC 100% CM (**D**), in PDLSC 50% CM (**E**), and in DPSC 50% CM (**F**). Graphs (**G**–**K**) indicate the different parameters analyzed in the tubulogenesis assay, with * *p* < 0.05, ** *p* < 0.01, *** *p* < 0.001, and **** *p* < 0.0001.

The analysis of the angiogenic potential showed that HMEC-1 cultured in endogrow medium formed a well-organized capillary network, as expected, as this medium is the standard pro-angiogenic one (Figure 4A), whereas HMEC-1 in α-MEM + 5% hpl (the growing medium of PDLSCs) were unable to form a precise structure (Figure 4B). Interestingly, HMEC-1 cultured in PDLSC and DPSC 100% CM (Figure 4C,D) and in PDLSC and DPSC 50% CM (Figure 4E,F) were capable of forming a complex capillary network, comparable to the one obtained when HMEC-1 were cultured in endogrow medium (Figure 4A). To better characterize the complexity of the capillary network, different parameters were evaluated. In detail, total length showed the maturation in space of the network (Figure 4G), while number of nodes, junctions, and segments highlighted the interconnection inside the network (Figure 4H–K). All together, these parameters put in evidence that there was no significant difference between HMEC-1 cultured in PDLSC 50% or 100% CM and DPSC 50% or 100% CM in terms of complexity of the network. Moreover, the conditioned media of the two cell types, with no distinction between 50% and 100% CM, had the capacity to induce the formation of a capillary network as well organized and complex as the one obtained when endothelial cells were seeded in endogrow medium. By contrast, the organization of the capillary network formed by HMEC-1 in α-MEM medium was significantly different from the ones obtained with the other media, highlighting how endothelial cells in this medium were not able to develop a structured network. These results indicate that both PDLSCs and DPSCs were able to trigger a very strong pro-angiogenic response in endothelial cells.

### 3.4. VEGF Release by PDLSCs and DPSCs

**Figure 5:**
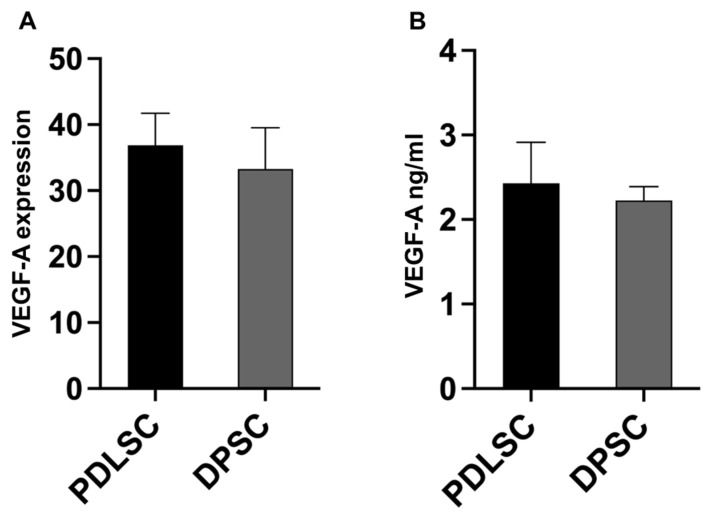
VEGF production. The VEGF relative gene expression was comparable between PDLSCs and DPSCs (**A**) as well as the VEGF quantified in CM (**B**).

The capability of PDLSC and DPSC CM to stimulate tubulogenesis was comparable to endogrow medium, suggesting that pro-angiogenic factors were released by both PDLSCs and DPSCs. Thus, we analyzed the gene expression of VEGF-A, showing that it was equally expressed by both PDLSCs and DPSCs (Figure 5A). Then, we quantified in PDLSC- and DPSC-conditioned media the presence of VEGF-A, which resulted as being comparable (Figure 5B). These results show that VEGF-A contributes to the pro-tubulogenic activity of PDLSCs and DPSCs.

### 3.5. PDLSCs and DPSCs Promote HMEC-1 Vessel-like Structures in Co-Cultures

**Figure 6:**
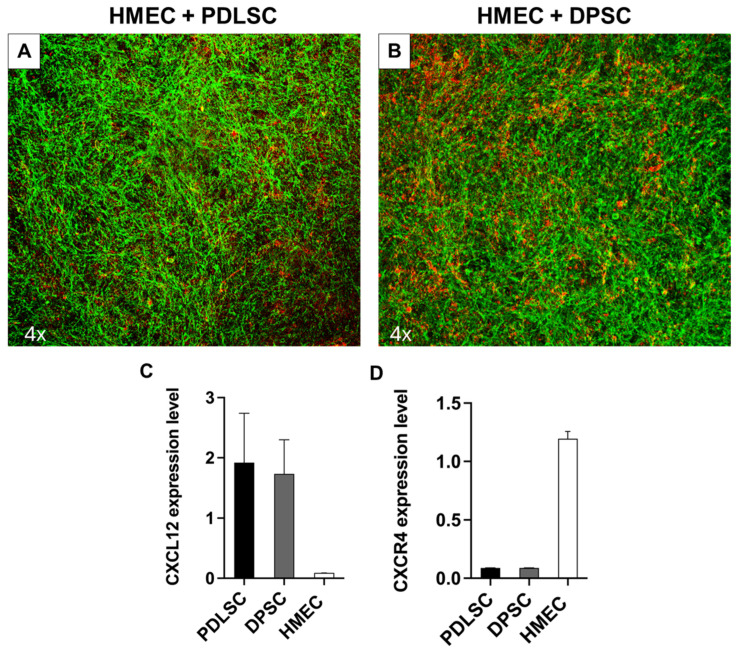
Co-cultures of H-MEC with PDLSCs and DPSCs. A network of CD31+ positive cells is visible in red, while PDLSCs and DPSCs cytoskeleton are in green (**A**,**B**). The histogram shows the relative expression of CXCL12 (**C**) and CXCR4 (**D**) by PDLSCs, DPSCs, and HMEC.

To further investigate the angiogenic potential of PDLSCs and DPSCs, we co-cultured them with HMEC-1 for 21 days, showing the co-existence of the different cells and the organization of HMEC-1 in a network of vessel-like structures (in red) below the sheet of PDLSCs and DPSCs (in green, Figure 6A,B). The CXCL12/CXCR4 pathway has been reported to be involved in the regulation of neovascularization during regenerative processes; thus, we evaluated the basal expression of these genes by PDLSCs, DPSCs, and ECs cultured alone. CXCL12 was expressed by both PDLSCs and DPSCs, while CXCR4 was expressed by HMEC-1 (Figure 6C,D), suggesting a reciprocal interaction between PDLSCs or DPSCs and HMEC-1.

## 4. Discussion

PDLSCs and DPSCs, soon after isolation, were a heterogeneous cellular population, expressing the typical MSC surface antigens, such as CD44, CD73, CD90, and CD105 [2], but also pericyte and endothelial markers [36]. The expression pattern of these markers is fluid, suggesting that these cells can respond to microenvironmental stimulation, triggering intracellular signaling that defines the cell phenotype. Our data are consistent with data in the literature reporting the expression of perivascular markers by both PDLSCs and DPSCs [15,37,38].

Notably, an increased subset of CD146+ cells was detected in DPSCs during culture, suggesting their plasticity and, likely, their ability to transition from the endothelial to mesenchymal phenotype and vice versa. The role of CD146 in angiogenesis and in vitro endothelial cell migration was reported [39]. It has also been described that the cross-talk between endothelial cells and DPSCs promote the maintenance of DPSCs themselves [40]. Moreover, DPSCs were able to switch to a vascular endothelial phenotype when transplanted in murine hosts [41], and due to the expression of VEGF receptor 1, DPSCs differentiate into endothelial cells more than bone marrow- and adipose-derived MSCs [27,28]. In PDLSCs, instead, a higher—even though not statistically significant—subset of CD31+ cells was detected, likely due to their origin; indeed, periodontium is largely vascularized, and pericytes lie close to endothelial cells, interacting with them to maintain and regulate the structure of blood vessels [42,43].

The heterogeneity of PDLSCs and DPSCs required an assessment of their stemness by analyzing the expression of the three well known landmarks—SOX-2, OCT3/4, and NANOG—which showed a similar expression in both the cell types. The presence of this stem cell pool is fundamental to guaranteeing tissue self-renewal, since stem cells interact with perivascular stem cell niche to regulate tissue regeneration [40,44]. The analysis of the basal expression of the master genes involved in the commitment of the main mesenchymal tissues bone, cartilage, and adipose tissue showed that in both PDLSCs and DPSCs there were subsets of more committed cells. According to data in the literature, we believe that this commitment could depend on the culture condition; indeed, it has been reported that serum-free culture condition can modulate the multi-differentiation ability of both PDLSCs and DPSCs [45,46]. The multi-differentiation potential of PDLSCs and DPSCs has been previously and broadly described [47,48]; here, we detected a comparable capability to osteo- and adipo-differentiation, but we reported a reduced chondrogenic ability of DPSCs compared to PDLSCs. Chondro-masses showed a slight stain for aggrecan and a reduced gene expression of SOX-9 and COLL-2A1. This is partly in disagreement with Vasandan AB et al. [49] who, directly comparing the two cell types, attained analogous chondrogenic potential of PDLSCs and DPSCs. However, Lei at al [50] provided comparative data that is consistent with our research. Although principally aimed at investigating the effect of in vivo transplantation of PDLSCs and DPSCs on their multi-differentiation abilities, these authors also reported data on the expression of chondrogenic genes and proteins by PDLSCs and DPSCs before the transplant, showing a lower level of aggrecan and COLL-2 in DPSCs than in PDLSCs [50].

As, to the best of our knowledge, the literature lacks a direct comparison regarding the angiogenic potential of PDLSCs and DPSCs, we performed it, showing that PDLSCs and DPSCs exerted a comparable pro-angiogenic activity, since they equally promoted tubulogenesis of HMEC-1. Indeed, the CM of both PDLSCs and DPSCs stimulated HMEC-1 to organize in vessel-like structures. Interestingly, CM was able to induce tubulogenesis at levels comparable to endogrow, suggesting that it, remarkably, was enriched in pro-angiogenic factors. One of the main agents regulating vasculogenesis and endothelial cells is VEGF [51], and we detected both gene expression and release of VEGF at comparable levels in PDLSCs and DPSCs.

Our results are in accordance with previously published data; thereby, the ability of PDLSCs to induce vessel-like structures in HUVEC was previously demonstrated [52], proving to be more angiogenic than bone marrow-derived MSCs [53]. The angiogenic potential of PDLSCs has also been demonstrated in mice model by injecting PDLSCs and HUVEC alone or together, showing that vessel-like structures formed only when cells were co-injected [54,55].

Several Authors reported the secretion of pro-angiogenic factors such as VEGF, FGF, and PDGF, by DPSCs [27,56,57]. In particular, in a work comparing the content of pro-angiogenic factors in CM deriving from MSCs, isolated in different sites of the oral cavity, DPSC CM resulted the most enriched in VEGF, FGF, and HGF, but PDLSC CM was not evaluated [26,58]. The angiogenic potential of DPSCs was also assessed in vivo in a mouse model of ischemia [59] and, more recently, the analysis of single-cell RNA sequencing identified a specific subset of DPSCs endowed in pro-angiogenic capabilities [60].

Since numerous studies showed the importance of MSCs in the formation of a stable microvasculature network [61], we co-cultured PDLSCs or DPSCs with H-MEC-1 for 21 days, showing a strong growth of mesenchymal cells, but also the presence of CD31+ endothelial cells, organized in vessel-like structures, confirming the results of the tubulogenesis assay. Similar data were also obtained by another group [62]. Among the pathways involved in the cross-talk between MSCs and endothelial cells emerged the role of CXCL12/CXCR4, which has been specifically studied in both DPSCs and PDLSCs, showing its involvement in DPSC and PDLSC proliferation, differentiation, and migration [63,64,65]. CXCL12/SDF-1 stimulates the maturation of blood vessels and simultaneously induces the recruitment of CXCR4-expressing cells in hypoxic condition, due to tissue damage [66], but also of pericytes and smooth muscle cells to organize themselves into new blood vessels [67]. In PDLSCs, the overexpression of CXCL12 enhanced the angiogenic potential of PDLSCs [68]. In the present work, we assessed the gene expression of CXCL12 and CXCR4, showing CXCR4 expression from HMEC-1, while its ligand, CXCL12, was mainly expressed at comparable levels from PDLSCs and DPSCs. A similar expression pattern was reported for PDLSCs and for DPSCs analyzed singularly [54,69,70].

The combinatory gene expression of angiogenic factor and chemokine by PDLSCs and DPSCs enables the enhancement of angiogenesis and consequently tissue regeneration [71]. Indeed, for regenerative treatment, it is fundamental to identify new ways to promote vascularization, above all for tissue like dental pulp that has a limited blood supply of root canals through a small apical foramen. Zhu et al. showed that the simultaneous induction of VEGF and SDF-1α overexpression in DPSCs significantly promotes pulp-like tissue compared to using the single factors alone [70]. Although we are conscious that our results derive from a small sample size, the study design based on paired cell populations may help reduce the need for high numbers. Indeed, since the inter-individual variability is relevant, the availability of two cellular subsets, PDLSCs and DPSCs, deriving from the same tooth, allows us to reduce the “inter-donor” variability.

## 5. Conclusions

Our data derived from the analysis of paired PDLSCs and DPSCs, which are heterogeneous populations of cells, constituted of different cellular subsets, likely modulated by microenvironmental cues. The direct comparison of PDLSCs and DPSCs showed an equivalent pro-angiogenic activity on endothelial cells, enhanced by the contemporary expression of angiogenic and chemotactic factors. These results suggest that both PDLSCs and DPSCs could be utilized for regenerative purposes, being conscious of their pro-angiogenic potential, fundamental for effective tissue regeneration.

## Data Availability

Dataset available on request from the authors.

## Supplementary Materials

The following supporting information can be downloaded at: https://www.mdpi.com/article/10.3390/cells14120864/s1, Figure S1. Relative COLL-2A1 expression. The expression of COLL-2A1 resulted significantly reduced in DPSCs compared to PDLSCs. * *p* < 0.01.
