# Supplementary material for: The Pro-Angiogenic Potential of Periodontal Ligament Stem Cells and Dental Pulp Stem Cells: A Comparative Analysis
Source: Cells. 2025 Jun 8;14(12):864. doi: 10.3390/cells14120864 (PMC12191286; doi:10.3390/cells14120864)
Supplement: Supplementary file 1 [file cells-14-00864-s001.zip › cells-3643049-supplementary.pdf]

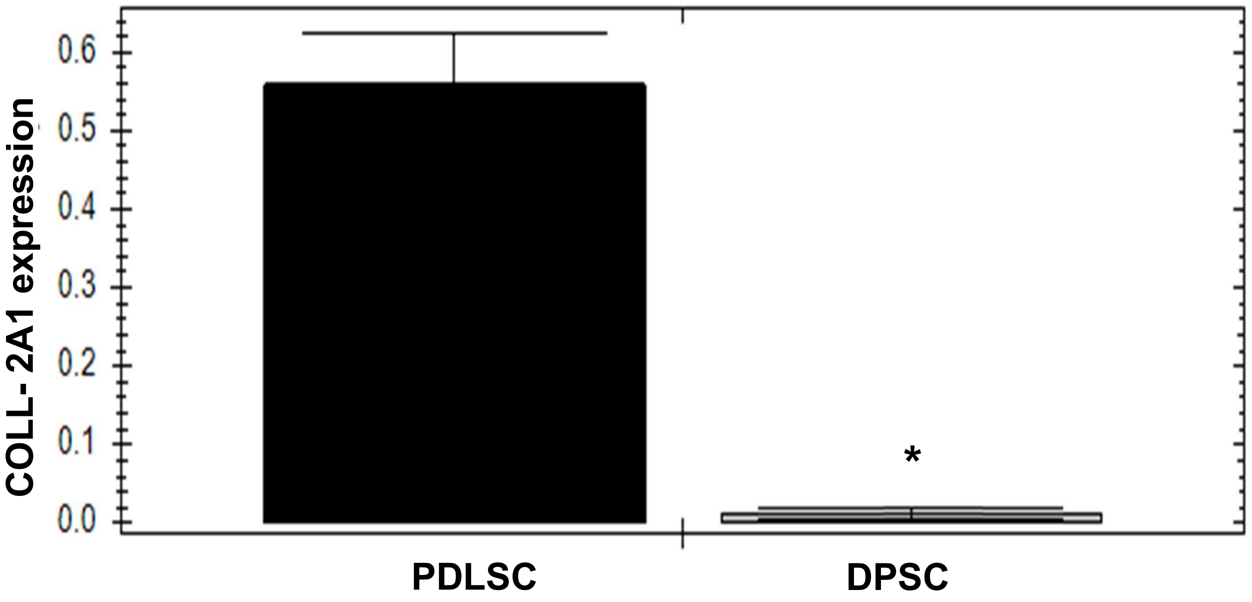

Figure S1. Relative COLL-2A1 expression. The expression of COLL-2A1 resulted significantly reduced in DPSCs compared to PDLSCs. * $p < 0.01$.
